# A Lightweight Pedestrian Detection Engine with Two-Stage Low-Complexity Detection Network and Adaptive Region Focusing Technique

**DOI:** 10.3390/s21175851

**Published:** 2021-08-30

**Authors:** Luying Que, Teng Zhang, Hongtao Guo, Conghan Jia, Yuchuan Gong, Liang Chang, Jun Zhou

**Affiliations:** School of Information and Communication Engineering, University of Electronic Science and Technology of China, Chengdu 611731, China; 201821010433@std.uestc.edu.cn (L.Q.); 202021010224@std.uestc.edu.cn (T.Z.); ght_work@163.com (H.G.); 201822010437@std.uestc.edu.cn (C.J.); 201911012135@std.uestc.edu.cn (Y.G.); liangchang@uestc.edu.cn (L.C.)

**Keywords:** pedestrian detection, lightweight, neural network, adaptive, FPGA

## Abstract

Pedestrian detection has been widely used in applications such as video surveillance and intelligent robots. Recently, deep learning-based pedestrian detection engines have attracted lots of attention. However, the computational complexity of these engines is high, which makes them unsuitable for hardware- and power-constrained mobile applications, such as drones for surveillance. In this paper, we propose a lightweight pedestrian detection engine with a two-stage low-complexity detection network and adaptive region focusing technique, to reduce the computational complexity in pedestrian detection, while maintaining sufficient detection accuracy. The proposed pedestrian detection engine has significantly reduced the number of parameters (0.73 M) and operations (1.04 B), while achieving a comparable precision (85.18%) and miss rate (25.16%) to many existing designs. Moreover, the proposed engine, together with YOLOv3 and YOLOv3-Tiny, has been implemented on a Xilinx FPGA Zynq7020 for comparison. It is able to achieve 16.3 Fps while consuming 0.59 W, which outperforms the results of YOLOv3 (5.3 Fps, 2.43 W) and YOLOv3-Tiny (12.8 Fps, 0.95 W).

## 1. Introduction

Pedestrian detection has been widely used in applications such as autonomous driving, video surveillance, and intelligent robots. In the past, the pedestrian detection was realized by traditional image processing and machine learning methods, such as the histogram of oriented gradient (HOG) and the support vector machine (SVM). The HOG is a classic feature descriptor used in image processing, which can effectively extract the features of pedestrians and send them to classifiers, such as SVM, to realize pedestrian detection [1]. Later, a discriminatively trained part-based models (DPM) detection method is proposed, which improves the detection accuracy by constructing the excitation template of each component, such as an edge and corner feature, then determines the target position according to the distribution of the excitation [2]. Wavelet domain-based pedestrian detection methods have also been proposed, for instance, a multi-feature (Multiftr) detection method is proposed, which extracts Haar-like wavelets features for the detection [3]. To further improve the detection accuracy, an aggregate channel features (ACF) detection method is proposed, which classifies aggregated channel features by decision tree [4]. As the features of pedestrians become more and more complex, such as appearance, clothing, dress, posture, lighting, background, and image resolution, it becomes increasingly challenging for the traditional methods to extract the effective features and achieve high detection accuracy. Also, it takes significant engineering effort to find suitable features for the detection.

To address the above issues, deep learning-based pedestrian detection methods have been proposed. Compared to the traditional methods, using signal processing and machine learning, these methods are able to extract the complex features of pedestrians more effectively and automatically through an end-to-end neural network, which leads to a higher detection accuracy and reduced effort for feature engineering. There are some scholars using a combination of machine learning- and deep learning-based methods. For example, in [5], a pedestrian method, which used SVM and CNN, is proposed. In this method, a motion detection method is used to locate the interested area, then principal component analysis and support vector machines are used to extract the textural feature vector and filter out interference regions, and, at last, a CNN is used to execute the pedestrian classification. Besides, many deep learning-based pedestrian methods have been proposed, such as active detection module (ADM) [6], multi-scale deep convolutional neural network (MS-CNN) [7], and graininess-aware deep feature learning (GDFL) [8]. These methods significantly improve the detection accuracy. In addition to the dedicated pedestrian detection methods, some methods are proposed by modifying the general object detection methods. For instance, pedestrian detection methods [9,10] based on YOLO and pedestrian methods [11,12] based on Faster R-CNN. However, while achieving a higher detection accuracy, the deep learning-based methods significantly increase the computational complexity, and cause high power consumption and a large processing time, making them unsuitable for hardware- and power-constrained mobile applications, such as drones and mobile robots.

In this work, we propose a lightweight pedestrian detection engine with a two-stage low-complexity neural network and adaptive region focusing technique, for power- and resource-constrained intelligent video surveillance applications, such as drone-based surveillance.

The main contribution of this work can be summarized as follows:

- A two-stage low-complexity neural network for pedestrian detection is proposed, which significantly reduces the number of parameters and operations of the detection neural network, while maintaining a high detection accuracy;
- An adaptive region focusing technique is proposed, which further reduces the computational complexity by removing the redundancy in the pedestrian detection in video streams;
- The proposed lightweight pedestrian detection engine has been implemented on a Xilinx FPGA Zynq7020, to evaluate its performance and power consumption.

## 2. Related Work

In the early stages, pedestrian detection was performed by extracting various features in the input image and sending them to a classifier for detection. For example, in [1], it is proposed to extract the HOG features of the image and use an SVM classifier for the pedestrian detection. To improve the accuracy, a latent SVM classifier is proposed and used with the HOG features for the pedestrian detection [13], which achieves a higher detection accuracy than using the conventional SVM classifier. In [14], it is proposed to combine the HOG features with the local binary pattern (LBP) features for the pedestrian detection. Other than the HOG-related methods, in [15], it is proposed to extract the gradient responses of the input image in different directions, and compute the local average of these responses around each pixel, to build shapelets features. The features are then sent to an Adaboost classifier for pedestrian detection. However, this method only relies on the edge features, which is not very efficient. In [16], the edge features are combined with the texture and color features to improve the detection accuracy.

In the recent years, deep learning-based methods have been heavily used in pedestrian detection, due to their high detection accuracy. Many works used the semantic information of a pedestrian for the detection. For example, the part and context network (PCN), using the semantic and contextual information of the body parts of the pedestrian [17]. Liu proposed a pedestrian detection method using the semantic labeling with the traditional HOG + SVM method, to improve the detection accuracy [18]. In pedestrian detection, the detection of small-scale pedestrians and occluded pedestrians are two major challenges. A pedestrian detection network (PedJointNet) is proposed that uses the head–shoulder feature of pedestrians as a complement to full-body prediction, to boost the detection accuracy [19]. In [20], a location bootstrap module and a semantic transition module are proposed to improve the detection accuracy of small-scale and occluded pedestrians. The location bootstrap is used to address predicted bounding boxes with relatively worse location precision, and the semantic transition module is used to extract more contextual information to relieve the semantic inconsistency of the skip-layer fusion for the detection of occluded pedestrians. Pedestrian detection in low-resolution images is also a challenge. In [21], a multi-resolution generative adversarial network (MRGAN) is proposed to simultaneously conduct multiresolution pedestrian detection, by generating high-resolution pedestrian images from low-resolution images. In [22], a fused discriminative metric learning (F-DML) approach is proposed to learn the optimal Mahanalobis metric, which transforms the low-resolution feature space into a new classification space, to improve the detection accuracy for low-resolution images. In addition, some works focus on pedestrian detection in low-light conditions. For example, a guided deep network is proposed to learn the extraction of multi-modal-like features from a single data modality in the framework of knowledge distillation via teacher–student training [23]. The teacher network, trained using the multi-modal data of RGB and thermal images, guides the student network to extract RGB and thermal-like features from RGB images alone. The multiScale detection network (MSDN) has also been proposed to solve the low-light problem [24]. In this method, a deep neural network is used to learn a non-linear mapping between RGB and thermal data. The learned feature representations are then transferred to another deep network, which receives the RGB image and generates the detection result.

Compared with the conventional machine learning methods, the deep learning-based methods achieve a higher detection accuracy. However, the computational complexity, including the number of parameters and operations, has been significantly increased. To address this issue, some low-complexity pedestrian detection methods have been proposed [25,26]. These works propose a lightweight neural network with a reduced number of parameters and operations. However, the reduction is still limited and/or the detection accuracy is heavily affected.

## 3. Proposed Pedestrian Detection Engine

**Figure 1:**
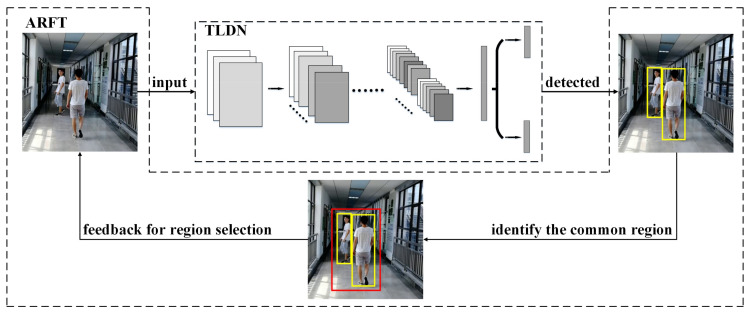
Overall architecture of the proposed method.

In this work, a low-complexity pedestrian detection method has been proposed for power- and resource-constrained intelligent video surveillance applications. In this method, a two-stage low-complexity pedestrian detection network (TLDN) is proposed, to reduce the number of parameters and operations in deep learning-based pedestrian detection. An adaptive region focusing technique (ARFT) is proposed to further reduce the number of operations, by utilizing the feature of pedestrian detection in a video stream. Figure 1 shows the overall architecture of the proposed pedestrian detection method.

### 3.1. Proposed Two-Stage Low-Complexity Pedestrian Detection Network

**Figure 2:**
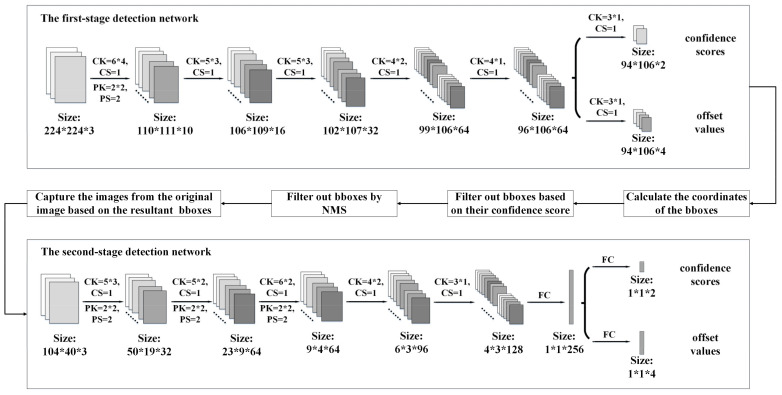
Proposed TLDN. The upper part is the first-stage detection network and the lower part is the second-stage detection network.

**Figure 3:**
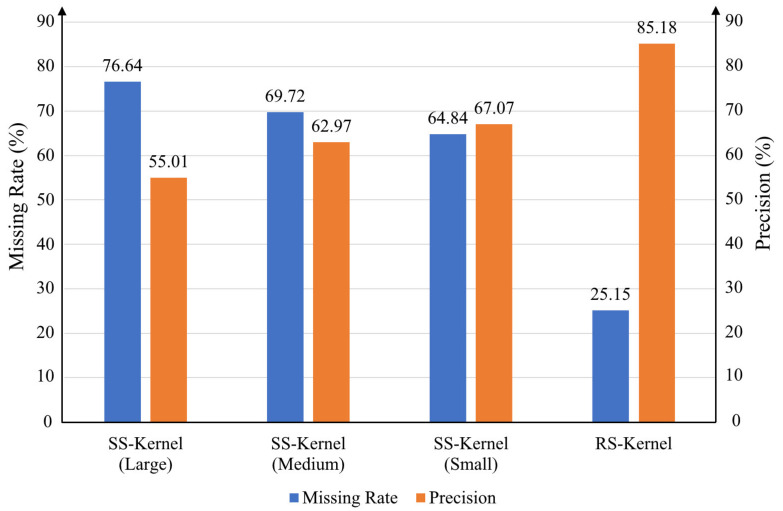
Comparison of experiment results of different kernels. SS-Kernel means square-shaped kernel and RS-Kernel means rectangular-shaped kernel.

The details of the proposed two-stage low-complexity pedestrian detection network are shown in Figure 2. The first stage of the detection network is used to generate the initial bounding boxes (bbox) of the pedestrians. Before the network, pyramid images are generated to obtain different scales of the input image. The first-stage network is a fully convolution network and, therefore, the size of the input image is flexible. As shown in Figure 2, the first-stage network contains only six convolution layers. As the network is shallow, in order to improve the efficiency of the feature extraction of pedestrians, rectangular-shaped kernels, instead of square-shaped kernels, are used for the convolution. We have conducted some experiments regarding the shape of the convolution kernel, as shown in Figure 3. It can be observed that with square-shaped convolution kernels, regardless of the kernel size, the precision is below 68% and the miss rate is above 64%. After changing the kernel shape to rectangular, the precision is improved to 85.18% and the miss rate is reduced to 25.15%. All the convolution layers in the first-stage network use rectangular-shaped kernels with different sizes, as shown in Table 1 and Figure 2, where CK, CS, PK, and PS are the convolution kernel size, convolution stride, max pooling kernel size, and max pooling stride, respectively. The outputs of the first network include the offset values of the obtained bboxes and their confidence scores.

With the offset values of the bboxes, the coordinates of the bboxes can be calculated. Firstly, the coordinates of the base bboxes can be calculated using Equations (1)–(4).

(1) $$x_{1}=\left(s_{w}*id_{x}\right)/r$$

(2) $$y_{1}=\left(s_{h}*id_{y}\right)/r$$

(3) $$x_{2}=\left(s_{w}*id_{x}+c_{w}\right)/r$$

(4) $$y_{2}=\left(s_{h}*id_{y}+c_{h}\right)/\;r$$

where $x_{1}$,$\;y_{1}$,$\;x_{2}$,$\;y_{2}$ are the horizontal and vertical coordinates of the upper left corner and the lower right corner of the base bboxes, respectively. $s_{w}$ is the ratio of the width of the input image to the width the output feature map, $s_{h}$ is the ratio of the height of the input image height to the height of the output feature map, r is the ratio of the size of the input image to the size of the largest scale pyramid image, $c_{w}$ is the allowed minimum width of the pyramid image, which is set to 15 (below this width, the pyramid image generation is stopped), and $c_{h}$ is the allowed minimum height of the pyramid image, which is set to 39. $id_{x}$ is the horizontal index of each pixel of the output feature map, and $id_{y}$ is the vertical index of each pixel of the output feature map.

After the coordinates of the base bboxes are calculated, the coordinates of the actual bboxes can be generated using Equations (5)–(8).

(5) $$x_{1}^{\prime }=x_{1}+\Delta x_{1}*\;\left(x_{2}-x_{1}\right)$$

(6) $$y_{1}^{\prime }=y_{1}+\Delta y_{1}*\;\left(y_{2}-y_{1}\right)$$

(7) $$x_{2}^{\prime }=x_{2}+\Delta x_{2}*\;\left(x_{2}-x_{1}\right)$$

(8) $$y_{2}^{\prime }=y_{2}+\Delta y_{2}*\;\left(y_{2}-y_{1}\right)$$

where $x_{1}^{\prime }$,$y_{1}^{\prime }$,$x_{2}^{\prime }$,$\;y_{2}^{\prime }$ are the horizontal and vertical coordinates of the upper left corner and the lower right corner of the actual bboxes after offset, respectively. ∆x1, ∆y1, ∆x2, ∆y2 are the offsets of the horizontal and vertical coordinates of the upper left corner and the lower right corner, respectively.

After that, the bbox selection is performed. First, some of the bboxes are filtered out based on their confidence score. A threshold is set to filter out the bboxes with a confidence score less than the threshold. Then, non-maximum suppression (NMS) is applied to remove the redundant bboxes, by selecting the bbox with the highest confidence score and calculating the intersection over union (IoU) value between it and all the candidate bboxes. The bboxes with IoU values higher than the threshold are removed. This process is iterated until no bbox can be removed.

The images corresponding to the resultant bboxes are sent to the second-stage network for computation one-by-one. The second stage of the detection network contains five convolution layers and two fully connected layers. All the convolution layers in the second-stage network use rectangular-shaped kernels with different sizes, as shown in Figure 2. The outputs of the second-stage network include the offset value of the obtained bbox and its confidence score for each image.

The second stage of the detection network is used to re-evaluate the initial bboxes. Its inputs are the images captured from the original image, based on the bboxes from the first stage. We will uniformly scale these images to 104 × 40, and then input them into the second-stage detection network. When the last layer of the second-stage detection network gets the output, we use Equations (5)–(8) to correct the bboxes represented by the input images.

The re-evaluated bboxes will be closer to the real situation. We filter all the re-evaluated bboxes, set a stricter score threshold, and then use the NMS algorithm to remove duplicate bboxes again. Through this stage of evaluation and filtering, we get the final pedestrian detection results.

### 3.2. Proposed Adaptive Region Focusing Technique

**Figure 4:**
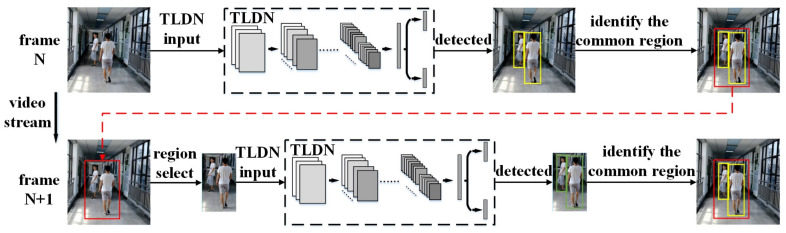
Proposed adaptive region focusing technique.

The proposed TLDN has small number of parameters; however, the second stage of the network needs to be computed multiple times, depending on the number of output bboxes from the first stage, which involves a large number of operations. To reduce the number of operations, we have proposed the ARFT technique, by utilizing the correlation among a sequence of frames in the video stream. The basic idea of the ARFT is to focus on the regions of the pedestrians in the image, and only perform the pedestrian detection in these regions for subsequent frames, to reduce the number of operations. The details of the technique are shown in Figure 4. When the pedestrians are detected in the input image, the regions of the pedestrians will be identified and used to guide the pedestrian detection for subsequent frames. A way to do this is to identify the regions for each detected pedestrian in the image and send them one-by-one to the detection network. However, this causes repeated computation of the detection network, especially when the number of regions is large, resulting in a limited reduction in the number of operations. To address this issue, we propose to identify the common region for all the detected pedestrians, as shown in Figure 4, and send it to the detection network for the subsequent frame. We first find a region that just includes all the bboxes of the detected pedestrians. Then, this region is enlarged by increasing its height and width outwards by 20%. This acts as a safeguard area to avoid a miss detection for next time, as the pedestrians may be moving. In this way, the detection network only needs to be computed once. Although the common region method slightly increases the region size compared to the separate region method, the final number of operations is significantly reduced (as shown in the experimental results in Section 5–D). In addition, to avoid the miss detection of newly appearing pedestrians outside the common region, a full-region pedestrian detection is performed intermittently (e.g., every five frames), or when no pedestrian is detected in the current frame.

## 4. FPGA Design

**Figure 5:**
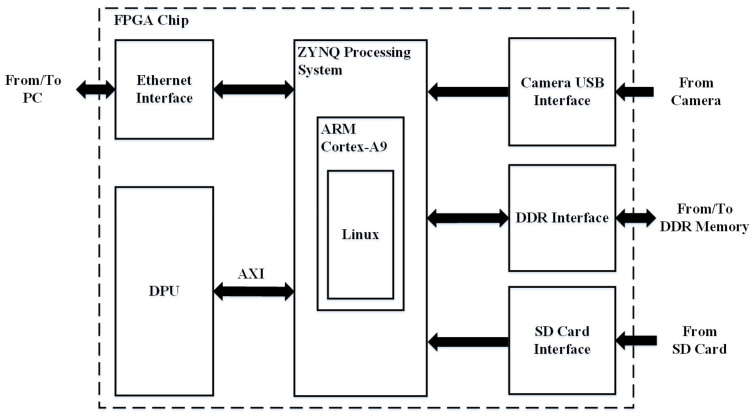
FPGA implementation.

The proposed pedestrian detection engine has been implemented on a Xilinx Zynq7020 FPGA, based on the deep learning processor unit (DPU). The DPU is an IP core provided by Xilinx for accelerating neural networks. It is able to support different neural network structures through reconfigurable hardware architecture (i.e., a neural network hardware accelerator including an array of processing engines, which are reused to execute the network layer-by-layer). As shown in Figure 5, the ZYNQ processing system based on the ARM Cortex A9 processor is used for realizing a Linux operating system, while the DPU is synthesized using FPGA logic as a hardware accelerator to communicate with the ZYNQ through AXI bus, for accelerating the neural network. During the operation, the main software program is executed partly in the Linux and partly on the DPU (the neural network part). The FPGA chip can communicate external peripherals, such as camera, PC, and SD card for data exchange. This forms a heterogeneous computing system for applications involving neural network computation.

**Figure 6:**
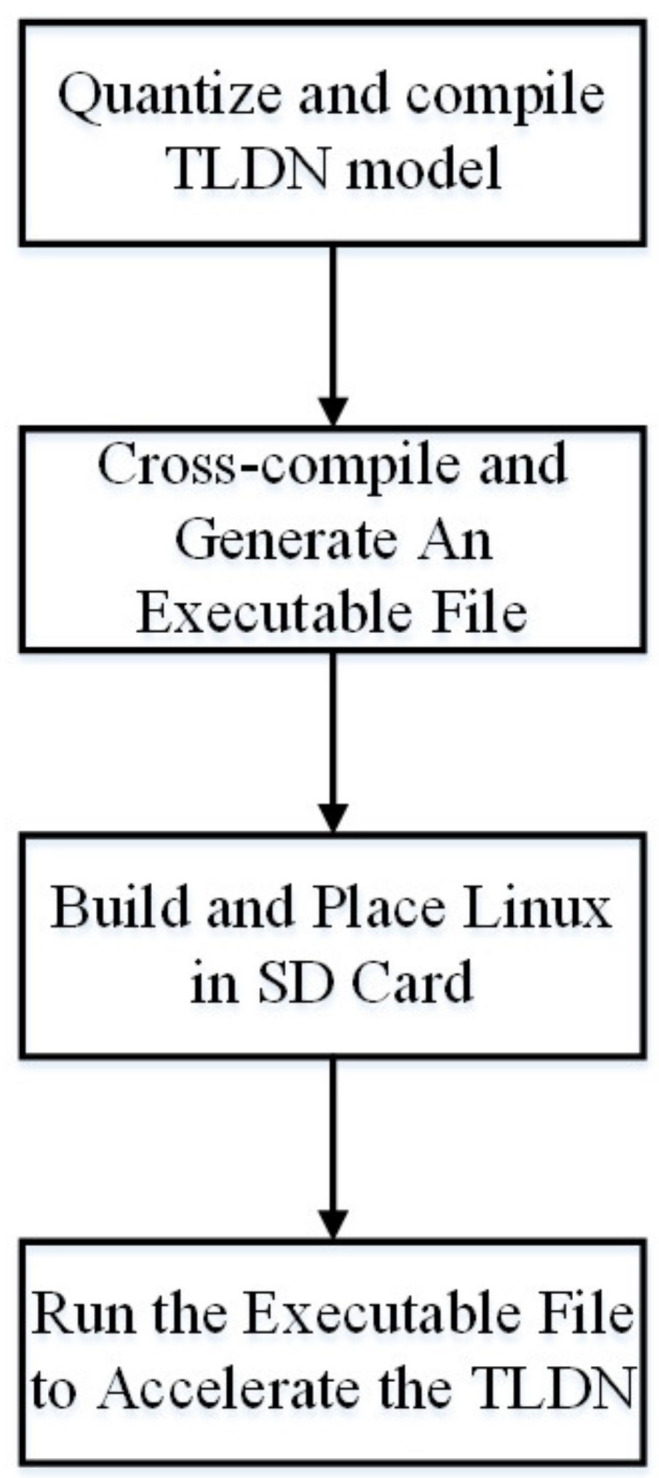
FPGA implementation flow.

The FPGA implementation flow is shown in Figure 6. First, the TLDN network model is quantized and compiled into DPU instruction codes by DNNDK, which is a toolchain provided by Xilinx. DPU is synthesized using FPGA logic as a hardware accelerator to communicate with the ZYNQ through AXI bus, for accelerating the neural network. Moreover, the inputs and outputs of the DPU unit are input nodes and output nodes of the TLDN network model. Then, a C++ program is written to initialize the DPU kernel, preprocess image, or video stream, before feeding them into the DPU task, processing on DPU, gaining the DPU output, and operating the postprocess on CPU. After completing the steps described above, the final result of the model will be gained. It also implements the algorithm to perform the ARFT operations, such as region identification and region selection. After that, the C++ program is cross-compiled together with the DPU instruction codes, which is included in the DPU driver provided by Xilinx, to generate an executable file to run in the Linux operating system. In the meantime, the root and kernel files for the ARM-based Linux operating system are built and placed in the SD card. During the operation, the root and kernel files are loaded into ZYNQ to start up the Linux operating system. Then, the executable file is run to execute the C++ program partly in the Linux and partly on the DPU (the neural network part), which calls the DPU instruction codes for accelerating the TLDN when needed.

**Figure 7:**
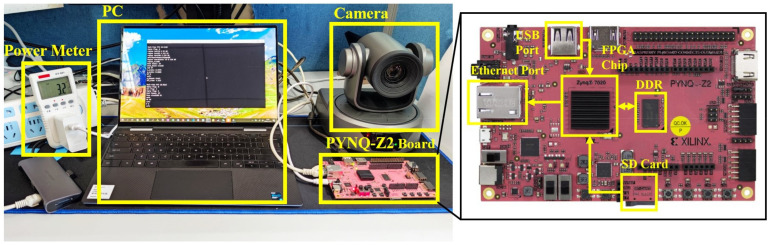
Experimental setup.

Figure 7 shows the test setup. A PYNQ-Z2 board is used for testing the speed and power consumption, which includes the Xilinx Zynq7020 FPGA chip, USB port, DDR memory, SD card, and Ethernet port. During the operation, the input video stream from a camera enters the system through the USB port. Based on the Linux system and OpenCv, the movie from the camera is read and preprocessed in the software before running the DPU task. The DPU implemented on the ZYNQ accelerates the TLDN layer-by-layer and exchanges intermediate feature map data with the on-board DDR. The pedestrian detection results are transferred to the PC through the Ethernet port, for monitoring. The architecture of the DPU is configured as B1152 (i.e., single DPU core), the system operating frequency is 140 MHz, and the resource utilization is shown in Table 2.

## 5. Experimental Results

Experiments have been conducted to evaluate the performance of the proposed pedestrian detection engine, and it has been compared with the existing pedestrian detection engines.

### 5.1. Training and Testing Dataset

The training dataset is built by combining a public dataset (i.e., Caltech dataset [27]) with a customized dataset. It includes more than 24,000 images from 110,000 pedestrians, under various scenes and illuminations. The training dataset is divided into the following three categories based on IoU: positive, partial, and negative, as shown in Table 3. The testing dataset is only the Caltech dataset, which includes 4024 images from 5051 pedestrians.

Specifically, for the training, the mixture of the Caltech dataset and a customized dataset is used, which includes only static images. For testing the accuracy, the Caltech dataset is used, which includes only static images. For testing the speed and power consumption, a camera is used to capture 10 min of movie (30 fps) as the input of the proposed network. This is able to evaluate the effect of the proposed adaptive region focusing technique.

### 5.2. Training

The training data of the first-stage detection network are generated as follows. First, random bboxes, with constraints on the width (15 pixels to half of the image width) and height (39 pixels to half of the image height), are generated for each image. Then, IoU is calculated between the generated bboxes and the label bboxes, to obtain the categories in Table 3. During the training of the first-stage detection network, the images with generated bboxes are cropped in the original images and resized to 39 × 15. Then, they are sent to the first-stage detection network in batches for training. After the first-stage detection network, the 39 × 15 images become 1 × 1, which corresponds to a set of classification and border regression values. The training data of the second-stage detection network are generated in a similar way, except that the bboxes are generated by the first-stage detection network instead of random functions and the cropped images are resized to 104 × 40. The base learning rate of the two-stage network training is set to 0.01 and the optimizer is a momentum optimizer.

The detection network has the following two outputs: the pedestrian/non-pedestrian classification result and the bbox regression. Therefore, a joint loss function is adopted for the training as follows:

(1) Pedestrian classification: Pedestrian classification is used to distinguish whether the image in the frame is a pedestrian or a background, so this is a two-classification task. We use the cross-entropy loss function for training. For each sample $x_{i}$, we use the following function:
  (9) $$L_{i}^{cls}=-\left(y_{i}^{det}\log\left(p_{i}\right)\;+\;\left(1-y_{i}^{det}\right)\left(1-\log\left(p_{i}\right)\right)\right)$$
  where $p_{i}$ is the network output for the sample $x_{i}$, which is used to indicate the probability that $x_{i}$ is a pedestrian. $y_{i}^{det}\in${0, 1} is from the ground-truth tag and represents the true value.
(2) Frame regression: Frame regression is used to reduce the position gap between the real frame and the predicted frame. Each frame includes the following four pieces of information: left border, upper border, height, and width. Therefore, we adopt Euclidean distance measure loss, as follows:
  (10) $$L_{i}^{box}=||{\hat{y}}_{i}^{box}-y_{i}^{box}||$$
  where ${\hat{y}}_{i}^{box}$ is the target frame obtained from the network output. $y_{i}^{box}$ is the real coordinate information, and it includes four dimensions, so $y_{i}^{box}\in R^{4}$.
(3) Joint loss function: since the network needs to complete two different tasks at the same time, it cannot use (9) or (10) alone as the loss function, so the joint loss function is introduced as follows:
  (11) $$L_{sum}=\sum_{i=1}^{N}\left(\lambda_{1}L_{i}^{cls}+\lambda_{2}L_{i}^{box}\right)$$
  where $L_{i}^{cls}$ is the loss function for the pedestrian classification, which uses the cross-entropy function, $L_{i}^{box}$ is the loss function for the bbox regression, which uses the Euclidean distance, and $\lambda_{1}$ and $\lambda_{2}$ are the weight coefficients for the two loss functions. For the first-stage detection network, we set $\lambda_{1}=1$ and $\lambda_{2}=0.5$. For the second-stage detection network, we set $\lambda_{1}=1$ and $\lambda_{2}=0.6$, to obtain more-accurate bbox coordinates.

### 5.3. Evaluation of Detection Accuracy

**Figure 8:**
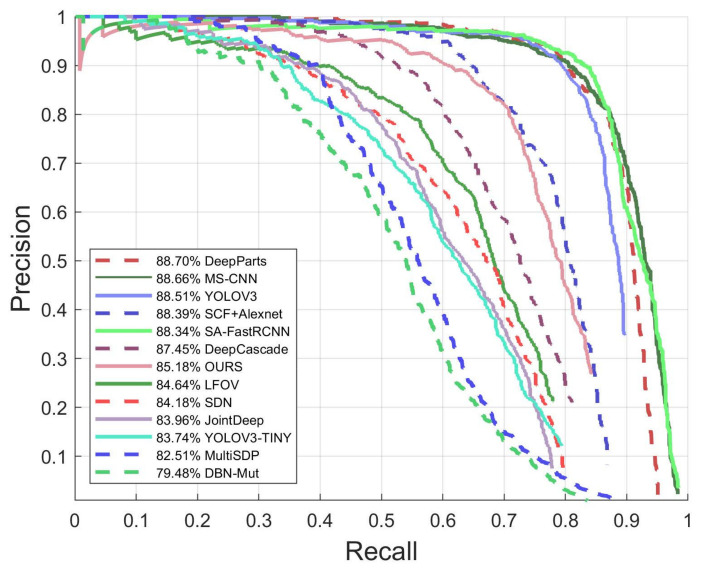
The precision–recall curve of the proposed method and the comparison with the existing methods.

**Figure 9:**
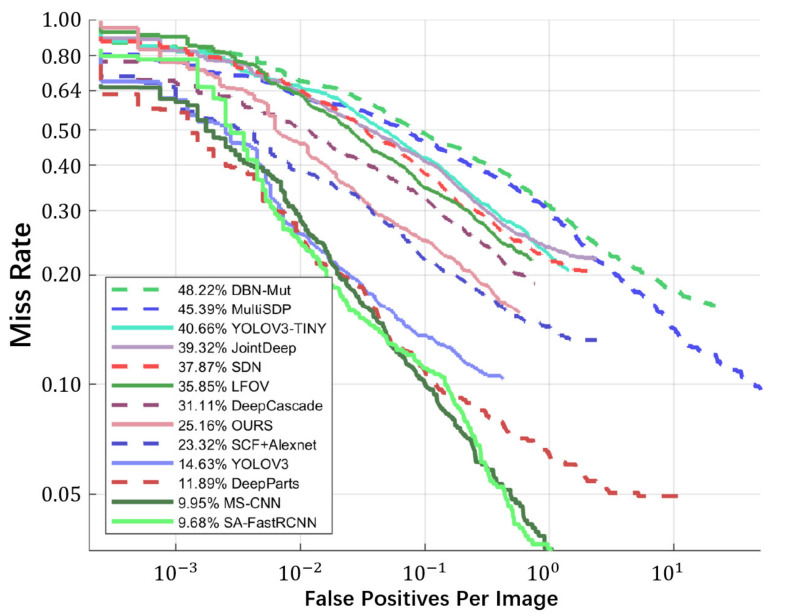
The miss rate curve of the proposed method and the comparison with the existing methods.

The detection accuracy is evaluated using precision, recall, miss rate and false positives per image, as shown in Equations (12)–(15). Figure 8 and Figure 9 show the precision−recall curve and miss rate curve of the proposed pedestrian detection engine. For comparison, we have evaluated some existing methods using the same testing dataset. As shown in Figure 8 and Figure 9, the proposed engine achieves a precision of 85.18% and miss rate of 25.16%, under the IoU threshold of 0.5, which are comparable to most of the existing methods.

(12) $$precision=\frac{true\;positives}{true\;positives+false\;positives}$$

(13) $$recall=\frac{true\;positives}{true\;positives+false\;negatives}$$

(14) $$miss\;rate=\frac{false\;negatives}{true\;positives+false\;positives}$$

(15) $$false\;positives\;per\;image=\frac{false\;positives}{the\;number\;of\;image}$$

### 5.4. Evaluation of Computational Complexity

Table 4 shows the computational complexity of the proposed engine and the comparison with the existing methods. Only the engines based on deep learning are listed here, as it is difficult to calculate the computational complexity of the engines based on conventional machine learning methods. The numbers of parameters and operations of deep learning-based engines are obtained by calculation using the neural network models provided in their papers. In Table 4, Ours-CR and Ours-SR both refer to the result of using a common region for the adaptive region focusing. It can be observed that our engine (Ours-CR) only requires 0.73 M parameters and 1.04 B operations. It outperforms the other engines in terms of the computational complexity, while achieving a comparable precision and miss rate. Compared to Ours-SR, Ours-CR has a reduced number of operations, due to the reduced number of computations of the detection network.

### 5.5. Evaluation of Speed and Power Consumption

Table 5 shows the measured power consumption and frame rate per second of the proposed engine for FPGA implementation. For comparison, we have also implemented YOLOv3 and YOLOv3-Tiny on the same FPGA board. However, DPU does not support the upsampling structure in YOLOv4 and v5, which is why we only implemented YOLOv3 and YOLOv3-Tiny for comparison. The power consumption is measured by using a power meter on the electrical socket of the FPGA board, as shown in Figure 7. As the FPGA board has a constant power consumption of ~2.6 W without any design implementation, this power consumption is removed during the measurement, for all the compared methods. It can be observed that the power consumption of the proposed engine achieves 16.3 FPS, while consuming 0.58 W on the PYNQ-Z2 FPGA board, which is significantly better than that of the YOLOv3 (5.3 Fps, 2.43 W) and YOLOv3-Tiny (12.8 Fps, 0.95 W).

## 6. Conclusions

In this paper, we propose a lightweight pedestrian detection engine with a two-stage low-complexity detection network and an adaptive region focusing technique, to reduce the computational complexity in pedestrian detection, while maintaining a high detection accuracy. Compared to the existing designs, the proposed pedestrian detection engine significantly reduces the number of parameters and operations, with a comparable precision (85.18%) and miss rate (25.16%). The proposed design has been implemented on FPGA for the evaluation of its real-time performance and power consumption. It is able to achieve 16.3 Fps while consuming 0.59 W, which is better than the mainstream detection engines, such as YOLOv3 (5.3 Fps, 2.43 W) and YOLOv3-Tiny (12.8 Fps, 0.95 W).

## Figures and Tables

**Table 1:** The values and specific names of CK, CS, PK and PS in every layer.

Stage	Layer	Convolution Kernel Size (CK)	Convolution Stride (CS)	Max Pooling Kernel Size (PK)	Max Pooling Stride (PS)
First stage	1	6 * 4	1	2 * 2	2
	2	5 * 3	1	/	/
	3	5 * 3	1	/	/
	4	4 * 2	1	/	/
	5	4 * 1	1	/	/
	6	3 * 1	1	/	/
Second stage	1	5 * 3	1	2 * 2	2
	2	5 * 2	1	2 * 2	2
	3	6 * 2	1	2 * 2	2
	4	4 * 2	1	/	/
	5	3 * 1	1	/	/

**Table 2:** Resource utilization.

	LUTs	Registers	DSPs	Block RAM
utilization	87,049	93,981	396	14,652 Kb

**Table 3:** Categories of training dataset.

Categories	IOU
Positive	≥0.65
Partial	≥0.4 ∩ <0.65
Negative	<0.3

**Table 4:** Computation complexity comparison of different methods.

Methods	Parameters	Operations	Precision	Miss Rate
OURS-CR	0.73M	1.04B	85.18%	25.16%
OURS-SR	0.73M	2.75B	85.18%	25.16%
SDN [28] ^2^	\	\	84.18%	37.87%
DeepParts [29] ^2^	187.10M	6.81B	88.70%	11.89%
LFOV [30] ^2^	135.27M	0.64B	84.64%	35.85%
SA-FastRCNN [31] ^2^	266.84M	41.35B	88.39%	9.68%
MS-CNN [7] ^2^	~217M	\	88.66%	9.95%
OR-CNN [32] ^2^	138.34M	30.94B	\	4.1%
ALFNet [33] ^2^	48.4M	5.07B	\	22.5%
CSP [34] ^2^	~31.23M	~67.03B	\	4.5%
CSANet [35] ^2^	~22.66M	~11.54B	\	3.88%
YOLOv3-Tiny ^3^	7.86M	5.56B	83.74% ^1^	40.66% ^1^
YOLOv3 [36] ^3^	61.57M	65.86B	88.51% ^1^	14.63% ^1^
YOLOV4 [37] ^3^	64.03M	62.25B	88.66% ^1^	10.21% ^1^
YOLOV5s ^3^	7.3M	17.0B	88.16% ^1^	25.65% ^1^
MDFL [38] ^2^	~276.69	~61.88B	\	31.46%
MultiSDP [39] ^2^	\	\	82.51%	45.39%
DBN-Mut [40] ^2^	\	\	79.48%	48.22%
SCF+AlexNet [41] ^2^	233M	727M	88.39%	23.32%

^1^ This accuracy is obtained by replication the work and testing it using the same testing dataset. ^2^ Pedestrian detection network. ^3^ Objection detection network.

**Table 5:** FPS and power of different methods ^1^.

Methods	FPS	Power (W)
OURS-CR	16.3	0.59
OURS-SR	8.6	0.68
YOLOv3-Tiny	12.8	0.95
YOLOv3 [36]	5.3	2.43

^1^ The input image size is 224 × 224.

## Data Availability

Not applicable.
